# The perception of youth health centres’ friendliness: does it differ between immigrant and Swedish-Scandinavian youths?

**DOI:** 10.1093/eurpub/ckaa077

**Published:** 2020-05-17

**Authors:** Mazen Baroudi, Miguel San Sebastian, Anna-Karin Hurtig, Isabel Goicolea

**Affiliations:** Department of Epidemiology and Global Health, Umeå University, Umeå, Sweden

## Abstract

**Background:**

Ensuring a good quality service and equal access according to need for all young people is a key objective of the Swedish health system. The aim of this study was to explore youths’ perception of youth health centres’ (YHCs’) friendliness and to assess the differences in perception between immigrant and Swedish-Scandinavian youths.

**Methods:**

All YHCs in the four northern counties in Sweden were invited (22 centres), and 20 agreed to participate. Overall, 1089 youths aged 16–25 years answered the youth-friendly health services-Sweden questionnaire between September 2016 and February 2017. Thirteen sub-domains of friendliness were identified and their scores were calculated. Multilevel analysis was used to examine the differences in perception between immigrant and Swedish-Scandinavian youths.

**Results:**

Our sample consisted of 971 Swedish-Scandinavian youths (89.2%) and 118 immigrants (10.8%). Generally, both groups perceived the services to be very friendly. All 13 sub-domains were rated more than three in a four-point scale except for fear of exposure and parental support of psychosocial services. However, immigrant youths perceived YHCs less friendly than their counterparts, particularly regarding the domains of equity, respect, quality and parental support.

**Conclusions:**

Our study suggests that even though youths perceived YHCs as highly friendly, there is a space for improvement regarding access to health care. Our findings highlight the importance of an open and culturally sensitive attitude of the staff and the need to engage parents and community as a key to improve immigrant youths’ accessibility to health care.

## Introduction 

The concept of youth-friendly health services (YFHSs) was first introduced by the World Health Organization (WHO) as health services that can engage youths and respond to their needs in sensitive and effective ways.^1^^,^^2^ These services should be perceived by youth as: (i) non-judgemental, sensitive and competent, (ii) providing services with respect and confidentiality, (iii) known and accepted by the youth and (iv) supported by the community.^3^^,^^4^ Moreover, YFHSs should ensure equal care to all regardless of their socioeconomic backgrounds.^4^^,^^5^ Access to YFHSs is a main strategy to ensure better sexual and reproductive health (SRH) and to reduce the unmet health needs among young people.^1^^,^^2^

Youth health centres (YHCs) in Sweden are differentiated health services directed to all youth aged 12–25 years. With ∼300 centres, these services are spread all over Sweden. Visiting YHCs is free of charge and all youth are entitled for this service including asylum seekers and undocumented immigrants.^5^ However, unequal access of this service by various youth groups has been suggested in the literature.^5^^,^^6^

Besides the heterogeneity of youths that goes beyond gender and place of residence,^7^ there is a growing ethnic diversity in Sweden. Currently, there are ∼2 million foreign-born residents in the country accounting for ∼20% of the population.^8^

The literature suggests that immigrants are facing disparities in accessing health care services, especially those related to SRH including YHCs.^5^^,^^6^^,^^9^^,^^10^ Various challenges are contributing to these disparities in access: first, factors related to immigrants’ conditions such as their socioeconomic status, education, social capital, health literacy, language skills and lack of knowledge about the health system. Second, those related to the health care provider such as cultural competence, miscommunication and racism. These factors might lead to poor access to health care services, lower quality of care and lower adherence to treatment, and therefore, to worse health outcomes among immigrants.^9^^,^^11–16^

Additional challenges should be considered regarding the access to YHCs in northern Sweden since this region spreads over half the area of the country but it is inhabited by only 10% of the population. These include the recruiting and retaining of health care staff hence ensuring the quality of the services, geographical distances and the difficulties to reach vulnerable populations, such as immigrant youths.^6^^,^^17^

YHCs in Sweden have been running since the 1970s and some reports suggest that they are well functioning^5^^,^^18^; however, no external evaluation has ever been conducted. Professionals working at YHCs have pointed out that inequities in terms of limited access for certain groups of youth might exist, specifically non-Swedish youths despite a dire need among this group.^6^ It is therefore important to explore if immigrant youths perceive the services different from Swedish Scandinavian youths.

Equity in health and health care has become a global priority, including Sweden.^19^ To reach such equity it is important to identify the challenges that groups in situation of vulnerability, such as immigrant youths, face in their access to health care services.^20^^,^^21^ It is, therefore, needed to have information about their perception of the health services. The aim of this study was to explore youths’ perception of YHCs friendliness and to assess the differences in perception between immigrant and Swedish-Scandinavian youths.

## Methods

### Population and data collection

All YHCs in the four northern counties in Sweden (Norbotten, Västerbotten, Västernorland and Jämtland) were invited to participate (22 centres). Data were collected between September 2016 and February 2017 from youths visiting 1 of the 20 centres which finally were involved in the study. The youth-friendly health services-Sweden (YFHS-Swe), questionnaire was self-administered and was handed out to the youths by the health professionals working in the YHCs. Overall 1089 participants aged 16–25 years answered the YFHS-Swe questionnaire in addition to other background questions.

YFHS-Swe questionnaire is a new instrument to measure youths’ perception of YHCs friendliness that has been previously validated in the Swedish context.^22^ YHCs-related variables were collected from official documents or directly from the centres.

### Measures

#### Outcome variables

Thirteen domains of friendliness were identified previously from the YFHS-Swe using exploratory and confirmatory factor analysis.^23^ These included: (i) ‘access contact’: the easiness to get contact with the YHC; (ii) ‘access SRH’: the easiness to get help related to SRH; (iii) ‘access psychosocial’: the easiness to get help related to social and mental health; (iv) ‘fear of exposure’: the perception of refraining from seeking help because of the fear that parents, teacher or other adults would find out about the visit; (v) ‘equity’: differences in attendance based on gender, ethnicity, religion, appearance, sexual orientation or disability; (vi) ‘equity with legal concerns’: differences in attendance based on legal or judicial status; (vii) ‘respect’: the way the youth was treated during the visit; (viii) ‘privacy and confidentiality’: the consultation was done in private and the youth trust that the staff will not breech the confidentiality; (ix) ‘no judgement’: the staff had an open, caring and unprejudiced attitude; (x) ‘quality of consultation’: the help received was up to the youth’s expectation; (xi) ‘quality of facility’: the quality of centre, waiting room and information leaflets; (xii) ‘parental support of SRH’ services’ use by the youths and (xiii) ‘parental support of psychosocial’ health services’ use by the youths. Each of these domains contained three or more questions ranked between 0 (very bad) and 4 (very good). (For a detailed list of the questions refer Supplementary material.)

#### Individual variables

##### 

‘Country of origin’, the youth were classified as Swedish-Scandinavian if the participants and both parents were born in Sweden or other Scandinavian countries; or immigrant if the youth or at least one of the parents were born outside Sweden/Scandinavia. ‘Age’ was grouped into 16–17, 18–19 and 20–25 years. ‘Gender’ was categorized into women, men and others and ‘sexuality’ into heterosexual; LGBTQ, including asexual and questioning; and don’t want to categorize myself sexually. ‘First visit’ included if it was the first visit to the YHC or not. ‘Type of appointment’ was classified into booked appointment, drop-in and other types of appointments. ‘Reason for consultation’ was categorized into sexual, psychosocial, physical or mixed. ‘Health worker profession’ included midwife; psychologist or social worker; nurse, doctor or dietician or they do not know, depending who had the youth during their current visit met.

#### YHCs-related variables

Different characteristics that might affect the service friendliness were collected. ‘Opening hours’ included open more or less than 20 hours week. ‘Location of the YHC’, if it was within a health centre, within a health centre but with separate entrance or separated from the health centre. ‘Ways of booking an appointment’, according to the number of ways available to book an appointment in the YHC, i.e. by telephone, Internet or onsite. ‘Drop-in days’ ranked according to the number of drop-in days per week. ‘Social media’, if the YHC have its own Facebook page, no page or a common page for all county’s centres. ‘LGBTQ certification’ (staff trained in how to receive and treat LGBTQ youths) included: yes, no or planned to be certified in near future. ‘Mental health competency’ was defined according to the availability of staff with special training in psychotherapy (yes/no) and the ‘number of professions’ available in the YHC, as a proxy for multidisciplinarity.

### Statistical methods

YHCs’ and participants’ individual characteristics were first described. The individual characteristics were reported in the total sample and stratified by ‘country of origin’. Then, the mean of questions that comprised each of the 13 dimensions of friendliness were calculated and presented in a radar graph for Swedish-Scandinavian and immigrant youths. Structural equation modelling was used to predict the score of each of the 13 factors (the method has been described elsewhere^23^). The score was then normalized to allow comparison between the factors. The mean of all the 13 factor scores was calculated and labelled as ‘Friendliness’.

Multilevel analysis was applied to adjust for any variation caused by the differences in YHCs-related factors. Thus, the relationship between country of origin and the perception of the 13 dimensions of youth friendliness were assessed using random-intercept linear mixed models. First, a model with only the main exposure, i.e. country of origin as an explanatory factor was fitted (model 1). In model 2 individual-level variables were included and in model 3, the YHCs-related variables added. Only the statistically significant variables from model 1 were included in models 2 and 3, and the results are reported as *β* coefficient and 95% confidence intervals (CIs). Variables were checked for multicollinearity based on the variance inflation factor (VIF) considering as acceptable a VIF of less than 10. Stata Version 15.1 (Stata Corp. Inc., TX, USA) was used for the analysis.

### Ethical consideration

Ethical approval was granted by the Regional Ethical Review Board Umeå (Drn: 2015-190-31O). A written informed consent was collected from all participants.

## Results

Our sample consisted of 971 Swedish-Scandinavian youths (89.2%) and 118 immigrant youths (10.8%). Table 1 shows the sample characteristics of those who visited the YHCs in Northern Sweden and of the YHCs. Women accounted for more than 90% of the visits to YHCs, however, proportionally more immigrant men than Swedish-Scandinavian men visited the YHCs. Around 85% of the youths considered themselves as heterosexual. The main reason for consultation was SRH issues (62%) and midwives were the main visited health providers (68%).

**Table 1:** Sample characteristics including individuals’ characteristics stratified by country of origin and health centres’ characteristics

	Total (*N* = 1089), *n* (%)	Swedish- Scandinavian (*n* = 971), *n* (%)	Immigrant (*n* = 118), *n* (%)
Individual level variables			
Age			
16–17	345 (32.7)	304 (32.4)	41 (35.7)
18–19	324 (30.7)	289 (30.8)	35 (30.4)
≥20	385 (36.5)	346 (36.9)	39 (33.9)
Gender			
Women	976 (90.9)	881 (92.1)	95 (81.2)
Men	91 (8.5)	70 (7.3)	21 (18)
Other	7 (0.7)	6 (0.6)	1 (0.9)
Sexuality			
Hetero	926 (85.9)	827 (86.1)	99 (84.6)
LGBTQ+	130 (12.1)	113 (11.8)	17 (14.5)
Not categorized	22 (2.0)	21 (2.2)	1 (0.9)
First visit			
No	914 (85.2)	823 (85.8)	91 (79.8)
Yes	159 (14.8)	136 (14.2)	23 (20.2)
Type of appointment			
Booked	800 (74.4)	721 (75)	79 (68.7)
Drop in	203 (18.9)	184 (19.2)	19 (16.5)
Other	73 (6.8)	56 (5.8)	17 (14.8)
Reason of consultation			
Sexual	623 (62.3)	559 (62.9)	64 (57.7)
Psychosocial	156 (15.6)	139 (15.6)	17 (15.3)
Physical	108 (10.8)	98 (11)	10 (9)
Multiple reasons	113 (11.3)	93 (10.5)	20 (18)
Health worker profession			
Midwife	711 (68.1)	643 (69)	68 (63)
Psychologist, social worker	161 (15.4)	140 (15)	21 (19.4)
Nurse, doctor, dietician	72 (6.9)	62 (6.6)	10 (9.3)
Don't know	100 (9.6)	91 (9.7)	9 (8.3)
Clinic level variables	Number of centres (*N* = 20), *n* (%)		
Opening hours			
>20	11 (55)		
<20	9 (45)		
Location of the clinic			
Within a health centre	2 (10)		
With separate entrance	2 (10)		
Separated	16 (80)		
Ways of booking an appointment			
1	1 (5)		
2	12 (60)		
3	7 (35)		
Drop-in days			
0	6 (30)		
1–2	10 (50)		
3–5	4 (20)		
Social media			
Own Facebook page	5 (25)		
No Facebook page	6 (30)		
Common Facebook page	9 (45)		
LGBTQ certification			
Certified	4 (20)		
Not certified	12 (60)		
Planned to be certified	4 (20)		
Mental health competency			
Yes	9 (45)		
No	11 (55)		
Number of professions			
1–3	9 (45)		
4 or more	11 (55)		

**Figure 1:**
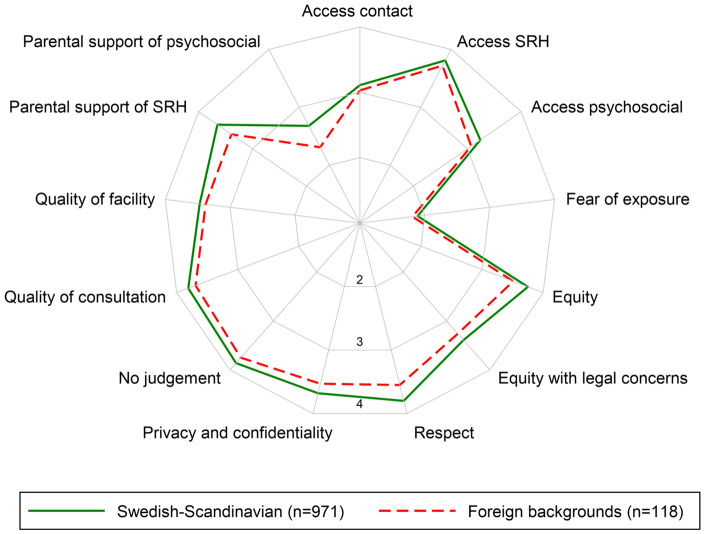
Differences in youth’s perception of the 13 dimensions of the youth health centre friendliness by country of origin

Figure 1 shows the differences in youth perception of the 13 youth-friendliness dimensions. Both immigrant and Swedish-Scandinavian youths perceived the YHCs to be very friendly (all rating more than three on a four-point scale) except in ‘fear of exposure’ and ‘parental support of psychosocial’ services. However, immigrant youths perceived YHCs slightly less friendly than Swedish-Scandinavian youths, particularly in the dimensions of ‘equity’, ‘respect’ and ‘parental support of SRH and psychosocial services’.

Table 2 shows the results of the multilevel analysis. According to the bivariate regression (model 1), seven outcomes were significantly lower among immigrant youth than among Swedish-Scandinavian youth. Those included: ‘equity, respect, no judgement, quality of consultation, parental support of SRH, parental support of psychosocial services’ and the overall friendliness.

**Table 2:** Perception of the dimensions of youth health centre friendliness among immigrant youths vs. Swedish-Scandinavian youths

	Model 1	Model 2	Model 3
	With country of origin, *β* (95% CI)	With individual variables, *β* (95% CI)	With individual and clinic-related variables, *β* (95% CI)
Access contact	−0.06 (−0.26 to 0.13)		
Access SRH	−0.16 (−0.36 to 0.04)		
Access psychosocial	−0.18 (−0.38 to 0.03)		
Fear of exposure	−0.15 (−0.36 to 0.05)		
Equity	−0.41 (−0.62 to −0.21)	−0.48 (−0.70 to −0.27)	−0.49 (−0.70 to −0.27)
Equity with legal concerns	−0.16 (−0.38 to 0.06)		
Respect	−0.46 (−0.66 to −0.26)	−0.48 (−0.70 to −0.26)	−0.48 (−0.70 to −0.27)
Privacy and confidentiality	−0.20 (−0.40 to 0.00)		
No judgement	−0.21 (−0.41 to −0.00)	−0.21 (−0.43 to 0.01)	
Quality of consultation	−0.23 (−0.43 to −0.02)	−0.27 (−0.49 to −0.05)	−0.27 (−0.49 to −0.05)
Quality of facility	−0.10 (−0.29 to 0.10)		
Parental support of SRH	−0.35 (−0.56 to −0.13)	−0.29 (−0.53 to −0.06)	−0.32 (−0.55 to −0.09)
Parental support of psychosocial	−0.30 (−0.52 to −0.08)	−0.31 (−0.55 to −0.07)	−0.31 (−0.55 to −0.08)
Overall friendliness	−0.35 (−0.54 to −0.17)	−0.42 (−0.63 to −0.22)	−0.43 (−0.63 to −0.22)

*β* (95% CI): *β* coefficient of the country of origin variable with 95% confidence interval.

After adjusting to individual factors (model 2), all statistically significant factors in model 1 except ‘no judgement’ continued to have statistically significant association with country of origin.

In model 3, after including the YHCs-related factors, all statistically significant factors in model 2 remained significant. Equity (*β*=−0.49; 95% CI: −0.70 to −0.27), respect (*β*=−0.48; 95% CI: −0.70 to −0.27), quality of consultation (*β*=−0.27; 95% CI: −0.49 to −0.05), parental support of SRH (*β*=−0.32; 95% CI: −0.55 to −0.09), parental support of psychosocial (*β*=−0.31; 95% CI: −0.55 to −0.08) and overall friendliness (*β*=−0.43; 95% CI: −0.63 to −0.22) were perceived worse among immigrant youths compared with Swedish-Scandinavian youths.

## Discussion

Our study shows similarities and differences between the immigrants and Swedish-Scandinavian youths’ perception of the YHCs’ friendliness. In general, both groups of participants perceived YHCs to be highly friendly. In addition, all youths perceived the possibility to get contact and help from the centres as good. However, the fear from being seen by parents or other adults was identified as a potential reason for refraining from visiting YHCs. The domains of equity, respect, quality of consultation and parental support of SRH and psychosocial health services were perceived to be lower among immigrant youths.

The YHCs in Sweden serve in general as a youth-centred primary health care service with a focus on health promotion related mainly to SRH. However, the care is based on a holistic approach that considers social, mental and physical aspects and on young peoples’ rights to information, support and equal and norm-critical treatment.^5^

Even though, the literature suggests that younger population tends to be less satisfied with health care services than older population,^24^ the highly positive perception of the YHCs shown in this study is in line with a previous evaluation of YHCs in Sweden.^18^

While the perception of how easy getting contact and help in the YHCs was perceived as good, health professionals working in these centres have expressed the existence of an unequal utilization of YHCs especially among immigrant youths.^6^ Several studies in Sweden and other European countries have also highlighted the lower utilization of preventive health care services, especially those related to SRH, among immigrants.^25–27^ Various factors might play a role in the lower immigrants’ utilization of the centres such as the lack of knowledge about the centres and their services including the knowledge about the eligibility for free services among both health care workers and immigrants, socio-cultural factors and language and communication difficulties.^10^^,^^15^^,^^25–28^ Our results highlight a lower perception of respect, equity and parental support which might also play a role in the lower immigrants’ utilization of the centres.

Similarly, the perception of the fear that parents or other adults would find out about the visit—which could lead to refraining from visiting YHCs—was identified equally, by both Swedish-Scandinavian and immigrant youths. Research has pointed out fear of confidentiality breach, particularly to parents, to be a main reason of refraining from health care services especially in sensitive consultations such as SRH or mental health.^29–31^ In one study conducted in the United States, 8% of the adolescents surveyed expressed un-met needs of health care due to fear that their parents would discover their visits.^31^

Five additional dimensions (equity, respect, quality and parental support to SRH and psychosocial services) were lower among immigrant compared with Swedish-Scandinavian youths.

Immigrant youths believed more that care was different due to discrimination based on gender, ethnicity, religion or other reasons. Providing equitable health services is essential to increase the acceptability and accessibility of such services.^4^ A review of governmental-run YFHSs in low- and middle-income countries showed that equity in care was the least addressed and assessed dimension of these services.^32^ Even though Swedish YHCs focus on equity as a means towards a quality care,^5^ these results stress the need of paying more attention to this dimension.

The study also revealed lower perception of being treated with respect among immigrant youths. Lack of respect has been shown to be the most common form of discrimination experienced by immigrants in health care in a study conducted in different EU states including Sweden.^15^ Often these disrespecting behaviours, which might be related to stereotypes, are perceived by the health care users as unfair rather than explicitly referring to it as discrimination or racism.^15^ Our results add to the existing knowledge that disrespectful treatment should be considered an important barrier facing immigrant youths.

Differences in the perceived quality of care between immigrant and Swedish-Scandinavian youths were also observed. Previous studies in other health care settings not youth related have shown evidence of a lower quality of health care provided to immigrants. For instance, lower satisfaction of post-natal care, inadequate medication and misinterpretation of cardiotocography suggesting suboptimal perinatal care for immigrants in Sweden has been reported.^33^^,^^34^

Finally, this study showed that both groups of youths perceived parents’ support of psychosocial services lower than parents’ support of SRH services. In both services, parents’ support was lower among immigrant youths than Swedish-Scandinavians. Parental support has been shown to play a positive role in youths’ adaptation of healthy behaviours such as physical activity and a protective role against risky health behaviours such as unsafe sex.^35^^,^^36^ Help-seeking behaviours are also connected to parental support. Youths from cultures that consider premarital sex as taboo and stigmatize mental health problems are more likely to turn to trusted friends or sibling for help instead of to the health care.^4^

### Methodological considerations

The study used an exit survey applied to youths after their visit to a YHC. While this method can give an accurate view of youths’ perception of the services provided, the potential lack of time and privacy are a challenge.

Our sample consisted of ∼11% immigrant youths including first and second-generation immigrants. There is no possibility for direct comparison with a corresponding proportion of youths in northern Sweden as data about second generation immigrants are not easily available. However, non-Scandinavian first- and second-generation immigrants make up ∼25% of the Swedish population.^8^ The lower representation of immigrant youths in our sample might be partially attributed to selection and participation bias as the questionnaire was administered by the YHCs’ staff and in Swedish. However, this lower representation probably suggests as well, the immigrant youths’ lower utilization of the health service.

Even though our sample might be representative of the YHCs’ users, it is not of the Swedish population. Moreover, the geographical restriction of our study to Northern Sweden might limit the generalization of the findings.

Youths from Scandinavian origins were joined with Swedish youths because of the similarity in Scandinavian languages, cultures and history of immigration.^37^ In addition, the similarity of health care system between Scandinavian countries might facilitate their access as they might be already familiar with the system.^38^ Thus, no important differences in perception between these groups would be expected. Regarding immigrant youths, those born outside Scandinavian were merged with those born in Sweden with one or two parents born outside Scandinavian in one category. This has been frequently used by authors looking into immigrants’ access to health care since both first- and second-generation immigrants might face similar challenges in access to health care.^39^

## Conclusions and recommendation

Our study shows that both immigrant and Swedish-Scandinavian youths perceived YHCs as highly friendly and easy to get contact and help in. However, both groups identified fear of being exposed to parents and other adults as a possible reason of refraining from seeking help. The study also shows that immigrant youths have lower perception of equity, respect, quality and parental support than the Swedish-Scandinavian youths.

To achieve equity in health care and to improve immigrant youths’ access of health services, policies and practices against discrimination and racism, which might be systematically embedded in the system, should be ensured. Additionally, there is a need to develop awareness and an open and sensitive attitude among the staff and to improve their communication with the youths. This could help bridging the cultural barriers and improving the perception of the service quality. Further measures could be taken to ensure that visiting the YHCs is kept in private, such as separate entrances for the centres and shorter waiting times in the waiting room. Finally, seeking support from parents and other community members such as teachers or school counsellors could be a relevant strategy to improve youths’ accessibility to health care.

The previous measures, corresponding to the challenges identified by our findings, could help to further improve the service of the already highly friendly YHCs in Sweden and for similar initiatives/services in other settings.

## Funding

This work was partly supported by the Swedish Research for Health, Working Life and Living Conditions (FORTE) [Grant Number 2014-0235].

*Conflicts of interest*: None declared.

**Key points**

- The youth health centres in northern Sweden are perceived as highly friendly services.
- Immigrant youths have lower perception of equity, respect, quality and parental support than the Swedish-Scandinavian youths.
- Both immigrant and Swedish-Scandinavian youths identified fear of being exposed to parents and other adults as a possible reason of refraining from seeking help in the centres.
- Several measures can be taken to achieve a more equitable health care; this includes developing an open and culturally sensitive attitude of the staff, improving their communication skills and ensuring the privacy of the visits.
- Engaging parents and community could serve as a key to improve immigrant youths’ accessibility to health care.

## Supplementary Material

ckaa077_Supplementary_Data
