# Supplementary material for: The perception of youth health centres’ friendliness: does it differ between immigrant and Swedish-Scandinavian youths?
Source: Eur J Public Health. 2020 May 17;30(4):780–5. doi: 10.1093/eurpub/ckaa077 (PMC7445032; doi:10.1093/eurpub/ckaa077)
Supplement: ckaa077_Supplementary_Data [file ckaa077_supplementary_data.docx]

Supplementary material: Questions related to each domain of the youth-friendly health services-Sweden (YFHS-Swe) questionnaire

| *Access contact* | *What do you think of the…*   1. Opening hours of this service? 2. Possibility of getting a booked appointment here? 3. Time you had to wait in the waiting room? 4. Possibility to be in contact, by phone, with this service? 5. Possibility to be in contact with the staff here (personally or by phone)? |
| --- | --- |
| *Access SRH* | *Do you think you would be able to receive help at this clinic related to…?*   1. Questions about or suspicions of pregnancy 2. To test for sexually transmitted infections 3. Questions about contraceptives |
| *Access psychosocial* | *Do you think you would be able to receive help at this clinic related to…?*   1. Mental problems, for example, worry, stress, depression, anxiety… 2. Questions concerning sexual orientation or gender identity 3. Concerning relationship to friend/partner 4. Smoking or help to stop smoking cigarettes 5. Problems with alcohol 6. Problems with marijuana or other drugs 7. Problems with parents or family 8. Problems with work/unemployment, school or university 9. Questions about food, exercise or sleeping habits 10. Concerning sexual actions that have occurred against your will 11. Concerning that someone has, or tried to, hurt you, or that you did it to someone else, for example fights, hits or kicks 12. Concerning that someone made you feel bad with something they said, for example threatened or insulted you |
| *Fear of exposure* | *Do you think a youth would abstain from seeking help on this service because…*   1. They are afraid that their parents would find out 2. They are afraid that teachers, staff or the principal would find out 3. They are afraid that the police would find out 4. They are afraid that their employer would find out 5. They are afraid that their friends would find out |
| *Equity* | *Do you believe that all groups of young people, have the possibility to receive the health care they are entitled to at this clinic?*   1. Regardless of their gender identity 2. Regardless of their ethnical background 3. Regardless of their social background 4. Regardless of their religion 5. Regardless of what they wear (clothes and appearance) 6. Even if they live on the street 7. Because of a certain sexual orientation 8. Even if they have a disability 9. Even if they have mental illness 10. Because they use drugs |
| *Equity with legal concerns* | *Do you believe that all groups of young people, have the possibility to receive the health care they are entitled to at this clinic?*   1. Even if they are violent 2. Even if they are involved in selling/buying sex 3. Even if they are in Sweden without legal permit |
| *Respect* | 1. How were you treated by the staff that you made the visit to today? 2. During the time of your visit, how comfortable did you feel? 3. How much trust do you have in this person? |
| *Privacy and confidentiality* | 1. How certain are you that the ones working in this service will not talk about your problems with other adults, if you don´t agree to it? 2. How much do you trust that the staff will keep your problems confidential? 3. Conversations with the staff are conducted in a way that no one else can hear what you are talking about? 4. Examinations are conducted in a way that no one else can see or hear you? 5. Did the staff ask you if you wanted to be alone, without the presence of a parent or another adult (answer only if you came accompanied by an adult)? |
| *No judgement* | *Grade the statements…*   1. The staff gave you their full attention 2. The staff respected your opinions and decisions 3. The staff treated you in a supporting and caring way 4. The staff had an unprejudiced attitude towards you |
| *Quality of consultation* | *Grade the statements…*   1. You received treatment or help that met your expectations 2. The staff explained to you, with words you could understand 3. The time that was allowed was enough for all your questions |
| *Quality of facility* | *Grade the following…*   1. The waiting room and the clinic facility? 2. Was there information material concerning youths’ health? 3. The quality of the information in the information material? |
| *Parental support of SRH* | *Would your parents or another important adult advise you to seek help at this clinic for these problems…?* (same as in ***access to SRH***) |
| *Parental support of psychosocial* | *Would your parents or another important adult advise you to seek help at this clinic for these problems…?* (same as in ***access psychosocial***) |
